# Lymphocyte-Directed Immunomodulation Remits Thymoma-Associated Autoimmune Pneumonitis

**DOI:** 10.1007/s10875-024-01760-3

**Published:** 2024-07-01

**Authors:** Elise M. N. Ferré, Diana X. Nichols-Vinueza, Lindsey B. Rosen, Peter D. Burbelo, Kevin P. Fennelly, Joseph Pechacek, Daniel M. Goldstein, Anahita Agharahimi, Annapurna Saksena, David E. Kleiner, Yesim Yilmaz Demirdag, Arun Rajan, David S. Schrump, Steven M. Holland, Alexandra F. Freeman, Michail S. Lionakis

**Affiliations:** 1 Laboratory of Clinical Immunology and Microbiology, National Institute of Allergy and Infectious Diseases (NIAID), National Institutes of Health (NIH), Bethesda, MD USA; 2 National Institute of Dental and Craniofacial Research (NIDCR), NIH, Bethesda, MD USA; 3 Pulmonary Branch, Division of Intramural Research, National Heart, Lung, and Blood Institute (NHLBI), NIH, Bethesda, MD USA; 4 Laboratory of Chronic Airway Infection, NHLBI, NIH, Bethesda, MD USA; 5 Laboratory of Pathology, Center for Cancer Research, National Cancer Institute (NCI), NIH, Bethesda, MD USA; 6 Department of Medicine, Division of Basic and Clinical Immunology, University of California of Irvine, Irvine, CA USA; 7 Thoracic and Gastrointestinal Malignancies Branch, NCI, NIH, Bethesda, MD USA; 8 Thoracic Surgery Branch, NCI, NIH, Bethesda, MD USA

**Keywords:** Thymoma, Azathioprine, Rituximab, Bronchiectasis, KCNRG, Pneumonitis

## Abstract

**Background:**

Thymoma presents with several autoimmune manifestations and is associated with secondary autoimmune regulator (AIRE) deficiency. Pneumonitis has recently been described as an autoimmune manifestation associated with thymoma presenting with similar clinical, radiographic, histological, and autoantibody features as seen in patients with inherited AIRE deficiency who suffer from Autoimmune PolyEndocrinopathy-Candidiasis-Ectodermal Dystrophy (APECED) syndrome.

**Objectives:**

To treat two patients with biopsy-proven thymoma-associated pneumonitis with lymphocyte-directed immunomodulation.

**Methods:**

Two patients with thymoma were enrolled on IRB-approved protocols at the NIH Clinical Center. We performed history and physical examination; laboratory, radiographic, histologic and pulmonary function evaluations; and measurement of the lung-directed autoantibodies KCNRG and BPIFB1 prior to and at 1- and 6-months following initiation of lymphocyte-directed immunomodulation with azathioprine with or without rituximab.

**Results:**

Combination T- and B-lymphocyte-directed immunomodulation resulted in improvement of clinical, functional, and radiographic parameters at 6-month follow-up evaluations in both patients with sustained remission up to 12–36 months following treatment initiation.

**Conclusion:**

Lymphocyte-directed immunomodulation remitted autoimmune pneumonitis in two patients with thymoma.

## Introduction

The thymus is essential for the development of T-lymphocytes [1]. Patients with thymoma have an abnormal thymic microenvironment and decreased AIRE expression associated with impaired negative selection of T-cells and a broad spectrum of autoimmune manifestations [2, 3]. Among those, myasthenia gravis is well-recognized to affect 30–44% of thymoma patients, but other autoimmune diseases have also been reported including Addison’s disease, erythroblastopenia, systemic lupus erythematosus, inflammatory myopathies, alopecia, vitiligo, thyroiditis, type 1 diabetes, hepatitis, pure red cell aplasia, pneumonitis, and Good’s syndrome [2, 4–8]. We previously reported that patients with thymoma-associated autoimmune pneumonitis exhibit similar clinical, radiographic, and histological features with autoimmune pneumonitis observed in the setting of Autoimmune PolyEndocrinopathy-Candidiasis-Ectodermal Dystrophy (APECED), also known as Autoimmune Polyendocrine Syndrome type 1 (APS-1), which is caused by loss-of-function *AIRE* variants [5, 9]. In addition, some thymoma patients carry autoantibodies against the lung-targeted bactericidal/permeability-increasing fold-containing B1 (BPIFB1), the potassium channel regulator (KCNRG), or both, which are also seen in APECED-associated pneumonitis [4, 5]. We previously demonstrated that lymphocyte-directed immunomodulatory treatment can remit APECED-associated autoimmune pneumonitis with improvement in clinical symptoms and radiographic and pulmonary function abnormalities [5]. The shared features between APECED-associated and thymoma-associated autoimmune pneumonitis suggest common immunopathogenic mechanisms. Thus, we postulated that the lymphocyte-targeted immunomodulatory regimen that remits APECED-associated autoimmune pneumonitis may also remit thymoma-associated autoimmune pneumonitis. Here, we describe the clinical presentation and response to lymphocyte-directed immunomodulatory treatment of two patients with thymoma-associated autoimmune pneumonitis.

## Methods

Patients provided written informed consent in accordance with the Declaration of Helsinki to participate in IRB-approved protocols NCT00001355 and NCT00018044 at the NIH Clinical Center. Pulmonary function testing (PFT) was performed in accordance with American Thoracic Society/European Respiratory Society standards [10]. Lung and liver tissue biopsies were routinely processed and sectioned for hematoxylin and eosin (H&E) and immunohistochemistry staining as previously described [5, 11]. A luciferase immunoprecipitation systems immunoassay was used to detect autoantibody immunoreactivity against BPIFB1 and KCNRG. A particle-based multiplex assay was used to detect immunoreactivity against anti-IFNα, anti-IFNβ, anti-IFNγ, anti-IFNω, anti-IL-12p70, anti-IL-17 A, anti-IL-17 F, anti-IL-22, anti-IL-23, and anti-GM-CSF. Seropositivity was determined based on the mean plus three standard deviations of control patients as previously described [5].

## Results

**Fig. 1:**
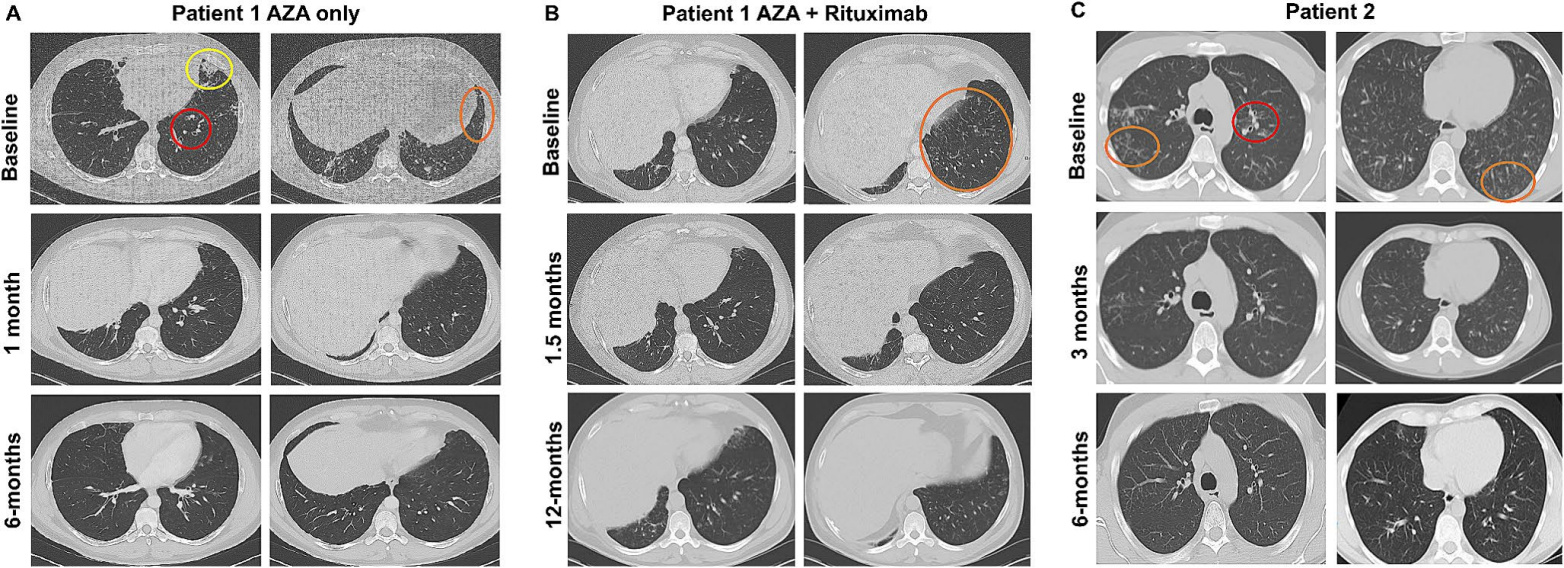
Radiographic, autoantibody, and histological features of thymoma-associated pneumonitis. Representative chest CT images for **A**) Patient 1 following azathioprine monotherapy at baseline and at 1-month and 6-months post-treatment initiation, **B**) Patient 1 following azathioprine with rituximab at baseline (before rituximab) and at 1.5-months and 12-months post-treatment initiation, and **C**) Patient 2 at baseline and at 3-months and 6-months post-treatment initiation. Baseline CT scans depict tree-in-bud nodularity (orange circles), bronchial wall thickening (red circles), and focal consolidations (yellow circle). Repeat CT scans demonstrate improvement at 1–3 months and resolution at 6-months following initiation of lymphocyte-directed immunomodulatory treatment. Note: Patient 1’s 1-month post-azathioprine monotherapy follow-up CT is complicated by right hemi-diaphragm elevation secondary to herpes zoster associated right phrenic nerve neuropathy. Abbreviations: AZA, azathioprine

**Fig. 2:**
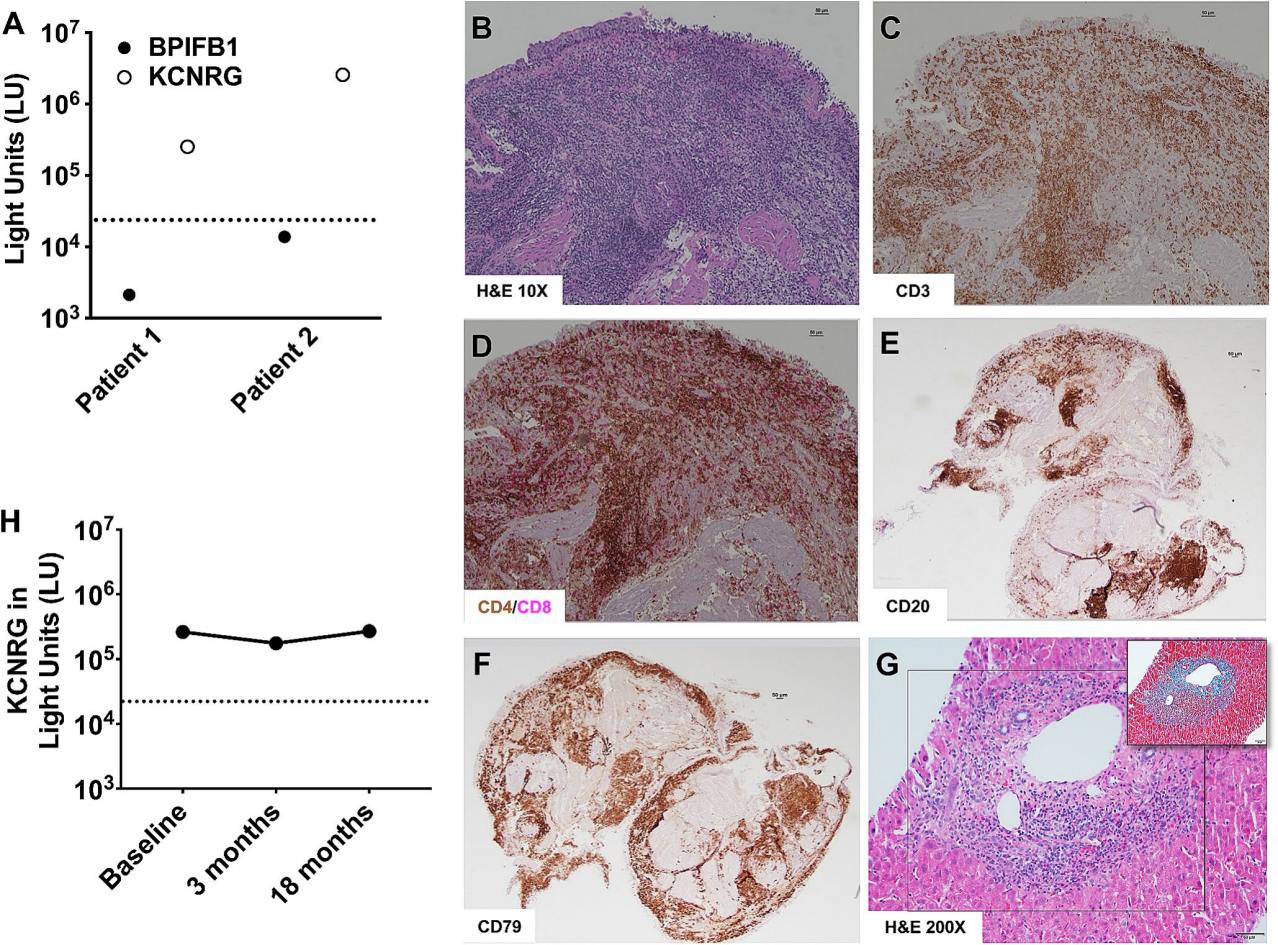
**A**) Autoantibody immunoreactivity against BPIFB1 and KCNRG presented as light units (LU). Dotted lines represent the cutoff values for determining autoantibody seropositivity. **B**) Hematoxylin and eosin (H&E) staining of endobronchial biopsy from patient 2 depicting chronic inflammatory infiltrate within intraepithelial and submucosal areas and thickened epithelial basement membrane. **C**) CD3 immunostaining showing that the infiltrate is composed primarily of CD3^+^ T lymphocytes. **D**) CD4 and CD8 double immunostaining showing that CD4^+^ T lymphocytes (brown) outnumber CD8^+^ T lymphocytes (magenta). **E**) CD20 immunostaining showing aggregates of B lymphocytes scattered throughout the lung parenchyma. **F**) CD79 immunostaining of the B cell receptor. **G**) H&E staining of the hepatic portal area of Patient 2 shows lymphocytic inflammation with scattered eosinophils and interface hepatitis. Masson trichrome shows mild periportal fibrosis (inset). Scale bars for panels B-G, 50 μm. Magnification for panels B-F, 10x. Magnification for panel G, 200x. H) Autoantibody immunoreactivity against KCNRG presented as LU in Patient 2 before treatment (baseline) and at 3- and 18-months following treatment with azathioprine and rituximab

The first patient is a 42-year-old male with a history of thymoma, nontuberculous mycobacterial (NTM) pulmonary infection, hypothyroidism, type 2 diabetes, and iatrogenic adrenal insufficiency who developed chronic cough productive of green sputum, dyspnea, pleuritic chest pain, and night sweats (Table 1). He was diagnosed at age 40 with a modified Masaoka stage 11 A, World Health Organization (WHO) type-B2 thymoma and bronchiolitis obliterans organizing pneumonia (BOOP; now known as cryptogenic organizing pneumonia) by lung wedge resection. He was treated with high-dose prednisone for BOOP. He was referred to the NIH for surgical resection where pre-surgical chest computed tomography (CT) identified bilateral lower lung infiltrates concerning for infection as well as bronchiectasis and tree-in-bud abnormalities throughout the lung fields. *Mycobacterium abscessus* grew from multiple sputum cultures. Thus, it became apparent that ‘BOOP’ was a misdiagnosis, and that the pathology was consistent with pulmonary NTM disease. Anti-mycobacterial therapy was initiated with azithromycin, linezolid, amikacin, and imipenem. One month later, he underwent right hemi-clamshell thoracotomy with resection of thymoma and pericardial reconstruction using Gore-Tex. Amikacin was discontinued secondary to mild ototoxicity, and clofazimine was added. Antibiotics were discontinued after 12 months of repeatedly negative mycobacterial sputum cultures and improved cough and radiographic abnormalities. He returned to NIH 3 months later with worsening respiratory symptoms and radiographic abnormalities concerning for recurrence of NTM infection. Sputum cultures grew *Mycobacterium avium complex* (MAC), *M. abscessus* with a different susceptibility profile from his initial isolate, and methicillin-susceptible *Staphylococcus aureus*. He improved symptomatically and sputum cultures became negative following 8-weeks of treatment with vancomycin, imipenem, azithromycin, and rifampin; however, his chest CT abnormalities of right middle and bibasilar focal consolidations, bronchial wall thickening, and nodular opacities (Fig. 1A) were largely unchanged despite antibiotics. He underwent bronchoscopy with bronchoalveolar lavage (BAL), which revealed airway neutrophilia (77% of nucleated cells) in the setting of negative microbiological studies including mycobacterial, bacterial, fungal, *Nocardia*, and *Legionella* cultures, respiratory viral FilmArray, PCRs for *Pneumocystis jirovecii*, *Legionella*, HSV, and CMV, and BAL *Histoplasma* and *Aspergillus* antigens. Histology demonstrated bronchial mucosa with chronic inflammation composed predominately of CD3^+^ T lymphocytes (CD4^+^ > CD8^+^ T lymphocytes) and aggregates of CD20^+^ B lymphocytes, a pattern also observed upon review of the initial lung wedge resection diagnosing thymoma (images previously published [5]). Autoantibody profiling identified high titers of KCNRG, but not BPIFB1, autoantibodies (Fig. 2A) as well as high titer anti-IFN-α and anti-IFN-ω (Table 2). Screening for autoantibodies against IFN-β, IFN-γ, IL-17 A, IL-17 F, IL-22, IL-23, and GM-CSF was negative (Table 2). Baseline pulmonary function testing (PFTs) revealed moderately severe mixed obstructive-restrictive ventilatory defect with FVC 79% of predicted, FEV_1_ 58% of predicted, FEV_1_/FVC 0.58, and a moderate diffusion defect [diffusing capacity of the lung adjusted for hemoglobin (DLCO_adj_), 60%] (Table 3). He had a 6-minute walk (6MWT) distance of 488 m, with 3% decrease in oxygen saturation.

**Table 1:** Summary of patients’ clinical characteristics

Demographics	Patient 1	Patient 2
Gender	Male	Male
Ethnicity	White	White
Age at presentation (years)	40	30
Thymoma treatment		
	Thymectomy without neoadjuvant / adjuvant chemotherapy	Thymectomy without neoadjuvant / adjuvant chemotherapy
Age of onset of autoimmune manifestations		
Alopecia barbae	n/a	30
Atrophic pancreatitis	n/a	30
Chronic active gastritis	n/a	30
Hepatitis	n/a	32
Pneumonitis	40	32
Hypothyroidism	41	n/a
Symptom description		
	Chronic cough productive of sputum, dyspnea, pleuritic chest pain, chronic sinusitis	Chronic cough, chronic sinusitis, steatorrhea, weight loss
Bronchoscopy findings		
	Scattered thick, white secretions throughout airways with numerous mucous plugs. Airways appeared mildly edematous and erythematous throughout.	Bronchial mucosa and anatomy were normal; there were no endobronchial lesions, and there were scant mucoid secretions.
Lung tissue histology		
Endobronchial biopsy	Bronchial mucosa with chronic inflammation. Predominately lymphocytic infiltrate in the lamina propria both singly and in aggregates made up of CD3/CD4 T-cells and CD20 B-cells and mixed with a few CD8 T-cells and CD4 histiocytes.	Respiratory Mucosa with dense chronic inflammation and thick epithelial basement membrane. CD20, CD3, CD4/CD8, CD79a and CD138 stains highlight reactive lymphoplasmacytic infiltrate.
Deeper lung biopsy	Bronchial tissue with peribronchial lymphocytic inflammation, scattered plasma cells and prominent infiltration of lymphocytes into bronchial epithelium. Foci of organizing pneumonia are seen at the edges of the peribronchial inflammation. There is a diffuse peribronchial infiltration of CD3 positive T-cells with CD4 positive T-cells outnumbering CD8 positive T-cells. Both B and T-cells infiltrate the bronchial epithelium. Small aggregates of CD20 positive B-cells are present around the edges of bronchi.	Respiratory mucosa with dense chronic inflammation and thick epithelial basement membrane. Acute and chronic inflammation associated with bronchial tissue. CD20, CD3, CD4/CD8, CD79a (B cell receptor), and CD138 (marker for terminally differentiated normal plasma cells) stains highlight reactive lymphoplasmacytic infiltrate.
Microbiology findings		
	Negative FITE and AFB stains. BAL negative for infectious organisms.	GMS stain negative for fungi and *Pneumocystis jirovecii*. BAL culture grew Mycobacterium intracellulare / chimaera
Immunosuppressive therapies		
T-cell targeting therapy	Azathioprine	Azathioprine
B-cell targeting therapy	Rituximab (1 gram x 2)	Rituximab (1 gram x 2)
Steroids	Prednisone	n/a

**Table 2:** Summary of patient 1’s radiographic and pulmonary function results at baseline and in response to azathioprine immunosuppression

	Baseline	1-months	6-months	1-year	2-year	3-year
Immunosuppression						
Azathioprine	100 mg	100 mg	100 mg	150 mg daily	150 mg daily	100 mg twice daily
Prednisone	5 mg	17.5 mg daily	17.5 mg daily	12.5 mg daily	12.5 mg daily	9 mg daily
Anti-mycobacterial therapy						
	Imipenem, azithromycin, rifampin, vancomycin	Imipenem, azithromycin, rifampin, vancomycin	Imipenem, azithromycin, rifampin, vancomycin	Imipenem, azithromycin, rifampin, vancomycin	Not applicable	Not applicable
6-Minute walk test						
Distance (m)	488	478	531	562	516	530
Pre O2	98	96	98	99	98	100
Post O2	95	98	99	100	98	98
Pulmonary function test						
FVC (liters)	4.01	3.62	3.77	4.63	3.31	4.15
FVC % predicted	79	71	72	89	64	81
FEV1 (liters)	2.33	2.38	2	2.36	2.12	2.76
FEV1% predicted	58	59	48%	57	52	68
FEV1/FVC%	58	66	53	51	64	67
DL Adj (mL/mmHg/min)	22	22.2	25.8	28.8	21.4	21.8
DL Adj % predicted	60	60	69	77	58	59
Spirometry Interpretation	Mixed pattern, moderately severe mixed obstructive-restrictive ventilatory defect; mild diffusion defect	Mixed pattern, moderately severe obstruction and restrictive ventilatory defect; mild diffusion defect	Moderately severe obstruction; mild diffusion defect	Stable, moderately severe obstruction, mildly reduced diffusion though improved from previous	Moderately severe obstruction with moderate restriction and moderately reduced diffusion	Mixed pattern with moderate obstructive and restrictive defect and moderate diffusion defect. Significant improvement in FEV1 and FVC since last study
Chest CT						
CT findings	Stable right hemithorax postoperative features. Right middle lobe and bilateral posterior lower lobe small focal consolidations and nodular infiltrates	Stable right hemithorax postoperative features. Decreased bibasilar tree-in-bud infiltrates	Stable right hemithorax postoperative features. Diffuse, minimal bronchiectasis with trace tree-in-bud opacities in bases	Stable right hemithorax postoperative features and bronchiectasis. Resolved bibasilar tree-in-bud opacities. Few scattered small ground-glass nodular foci in lungs bilaterally.	Stable right hemithorax postoperative features. Stable bronchiectasis	Stable right hemithorax postoperative features. Stable bronchiectasis

**Table 3:** Summary of patient 1’s radiographic and pulmonary function results at baseline and in response to combination azathioprine and rituximab treatment

	Baseline	1-months	1-year
Immunosuppression			
Azathioprine	100 mg	100 mg	100 mg
Prednisone	9 mg	9 mg	9 mg
6-Minute walk test			
Distance (m)	513	Not performed	537
Pre O2	98		98
Post O2	95		96
Pulmonary function test			
FVC (liters)	3.88	Not performed	3.98
FVC % predicted	76		86
FEV1 (liters)	2.22		2.35
FEV1% predicted	55		63
FEV1/FVC%	73		73
DL Adj (mL/mmHg/min)	21.4		21.24
DL Adj % predicted	58		72
Spirometry Interpretation	Mixed obstructive-restrictive pattern with moderately severe obstruction and mild restriction. Moderately reduced diffusion.		Moderate obstruction with mild diffusion defect.
Chest CT			
CT findings	Stable right hemithorax postoperative features. Scattered tree-in-bud opacities in bilateral bases	Stable right hemithorax postoperative features. Interval improvement in scattered bilateral tree-in-bud opacities	Stable right hemithorax postoperative features. Interval improvement in scattered bilateral tree-in-bud opacities

Before starting treatment for autoimmune pneumonitis, we tested for thiopurine methyltransferase (TPMT) activity to avoid the risk for azathioprine (AZA)-induced toxicity, which was normal [12]. We initiated AZA at 100 mg (1 mg/kg) daily and continued prednisone 20 mg (0.25 mg/kg) daily. Three weeks later, he developed herpes zoster infection presenting with a painful dermatomal rash affecting the right shoulder and extending to the neck, chest, and back and associated right phrenic nerve neuropathy resulting in right hemi-diaphragm elevation documented by chest X-ray and positive sniff test. He was treated with intravenous acyclovir while continuing azathioprine and prednisone was tapered to 17.5 mg daily (Table 3). At 1-month follow-up, he had improvement in cough, dyspnea, and chest CT findings of bibasilar infiltrates and tree-in-bud nodularity (Fig. 1A). By 6-months, his respiratory symptoms had resolved with further improvement noted in chest CT abnormalities (Fig. 1A). DLCO_adj_ improved to 69% and 6MWT distance increased to 531 m. At 1-year post-treatment initiation, attempts to wean prednisone resulted in recurrence of respiratory complaints. Consequently, over the next 1.5 years, AZA was titrated up to 100 mg twice daily allowing for tapering of prednisone to 9 mg daily, which he remained on for treatment of iatrogenic adrenal insufficiency. At 3-years post-treatment initiation, he remained respiratory symptom-free, had no radiographic abnormalities, and had stably improved PFT and 6MWT (Table 3).

Of interest, over the course of the next 6 months, he developed gradually worsening dyspnea, particularly upon climbing stairs. Chest CT (Fig. 1B) was notable for new scattered tree-in-bud opacities in the bilateral bases and sputum culture was positive for methicillin-susceptible *Staphylococcus aureus* for which he was prescribed a 14-day course of levofloxacin. Despite treatment, his symptoms did not improve, he continued to have radiographic abnormalities, and he demonstrated a severe mixed obstructive-restrictive spirometry pattern with moderate diffusion defect on PFTs in combination with a 6% decline in SpO_2_ on 6-minute walk test (Table 4). He underwent bronchoscopy with endobronchial biopsy for which histological examination revealed chronic inflammation composed of mixed CD4^+^ and CD8^+^ T lymphocytes and aggregates of B lymphocytes consistent with recurrence of autoimmune pneumonitis. We performed the same panel of microbiological studies on the BAL that were all negative. Given the treatment response we had observed in Patient 2 to combination T and B cell-targeted therapy and the favorable response with the combination of azathioprine and rituximab in APECED-associated autoimmune pneumonitis, we administered two intravenous infusions of rituximab (1 gram each) within 2 weeks and followed the patient clinically and radiographically at 1-month and 12-months post-treatment. By 1-month, he reported resolved cough and sputum production and improved dyspnea on exertion. Chest CT demonstrated clearing of tree-in-bud abnormalities in the bilateral bases. At 12-months, cough had resolved, and mild dyspnea occurred only when climbing *> 3 flights of stairs.* He demonstrated resolution of tree-in-bud abnormalities on CT and pulmonary function improvement on PFTs with FVC 86% of predicted, FEV_1_ 63% of predicted, and DLCO_adj_ of 72%.

**Table 4:** Anti-cytokine autoantibody profiles in the patients with thymoma presented in our study

	Patient 1	Patient 2
anti-IFNα	7963	8176
anti-IFNβ	41	197
anti-IFNγ	290	245
anti-IFNω	**8151**	**7871**
anti-IL-12p70	131	**8842**
anti-IL-17 A	68	151
anti-IL-17 F	183	178
anti-IL-22	104	195
anti-IL-23	24	110
anti-GM-CSF	86	38

Positive values are indicated in bold

The second patient is a 33-year-old male with no significant past medical history until age 20 when he was diagnosed and treated for guttate psoriasis, which resolved with UV therapy, and alopecia areata (Table 1). At age 30, he developed severe abdominal pain and weight loss. Abdominal CT revealed an atrophic pancreas and a lung mass. A transbronchial biopsy showed a WHO B1 thymoma and a pancreatic biopsy revealed atrophic pancreatitis with negative IgG and IgG4 staining. He underwent thymectomy and initiated pancreatic enzyme replacement, which resolved his abdominal symptoms. Following thymectomy, he developed persistent dry cough and recurrent sinusitis symptoms, which were unresponsive to several courses of antibiotics to treat *Pseudomonas* and MAC including rifampin, ethambutol, azithromycin, inhaled tobramycin, doxycycline, and levofloxacin for 6 months. Evaluation for vocal cord dysfunction and cystic fibrosis were negative. Autoantibody evaluations showed high titers of KCNRG, but not BPIFB1, autoantibodies (Fig. 2A) as well as high titers for anti-IFN-α, anti-IFN-ω, and IL-12p70 (Table 2). Screening for autoantibodies against IFN-β, IFN-γ, IL-17 A, IL-17 F, IL-22, IL-23, and GM-CSF was negative (Table 2). Chest CT revealed diffuse peribronchial thickening, mucus plugging, and tree-in-bud nodularity throughout the lungs (Fig. 1C). PFTs demonstrated a mild restrictive pattern (Table 5). He underwent bronchoscopy with BAL, which revealed airway neutrophilia (73% of nucleated cells). BAL cultures grew *Mycobacterium intracellulare/chimaera*. Endobronchial biopsies showed basement membrane thickening and profound lymphocyte infiltration in intraepithelial and submucosal areas. His lung biopsy showed chronic peri-bronchiolitis composed predominately of CD3^+^ T lymphocytes (CD4^+^ > CD8^+^ T lymphocytes) (Fig. 2B-F). Anti-mycobacterial therapy was started with azithromycin and ethambutol and inhaled amikacin was initiated 4 weeks prior to starting immunosuppression. During the first month of antibiotic induction period, routine surveillance labs noted persistently elevated transaminase levels for which he underwent liver biopsy that revealed chronic hepatitis with autoimmune hepatitis-like features of moderate activity with periportal and perivenular fibrosis such as that seen in APECED-associated autoimmune hepatitis (Fig. 2G) [11]. He received two doses of rituximab (1 gram each) within 2 weeks and, given normal TPMT activity, was started on AZA at 75 mg (1 mg/kg) daily. AZA was increased to 100 mg (1.3 mg/kg) once daily after one month of therapy (Table 5). At 3-months following AZA and rituximab initiation, the patient had no pulmonary complaints, had no radiographic abnormalities on chest CT imaging, had normalized transaminase levels, and demonstrated stability in PFTs and 6MWT. At 6-months, he remained asymptomatic with normal chest CT; due to the COVID-19 pandemic, he was unable to perform PFTs and 6MWT at that time. At 18-months after treatment initiation, he remained free of respiratory complaints, he had no radiographic abnormalities, he had normal transaminases and normal PFTs and 6MWT (Table 5), and his alopecia had resolved (Table 1). KCNRG antibodies remained highly positive despite rituximab administration (Fig. 2H) suggesting they may not be directly pathogenic [5]. He completed 18 months of treatment for MAC and remained on AZA 100 mg daily and has not necessitated re-dosing with rituximab.

**Table 5:** Summary of radiographic and pulmonary function results of patient 2 at baseline and in response to immunosuppression

	Baseline	3-months	6-months	18-months
Immunosuppression dosing				
Azathioprine	75 mg	100 mg	100 mg	100 mg
Anti-mycobacterial therapy				
	rifampin, ethambutol, azithromycin, inhaled tobramycin, doxycycline, and levofloxacin	rifampin, ethambutol, azithromycin, inhaled tobramycin, doxycycline, and levofloxacin	rifampin, ethambutol, azithromycin, inhaled tobramycin, doxycycline, and levofloxacin	rifampin, ethambutol, azithromycin, inhaled tobramycin, doxycycline, and levofloxacin
6-Minute walk test				
Distance (m)	716	719	Not performed	745
Pre O2	100	99		100
Post O2	98	99		100
Pulmonary function test				
FVC (liters)	4.59	4.43		5.19
FVC %	84	81	Not performed	95
FEV1 (liters)	3.43	3.44		4.24
FEV1%	78	78		96
FEV1/FVC%	68	78		82
DL Adj (mL/mmHg/min)	25.6	27.8		26
DL Adj %	64	70		65
Spirometry Interpretation	Borderline restrictive pattern. The flow volume loop has a mixed configuration; mild diffusion effect	Mild restrictive pattern. Mild diffusion effect.		Normal Flows. Normal total lung capacity using nitrogen washout technique. Mild diffusion defect.
Chest CT				
CT findings	Bilateral tree in bud nodules in the lung parenchyma. Nodular infiltrates in the right upper lobe. Diffuse scattered mild tubular bronchiectasis bilaterally.	Bilateral mild tubular bronchiectasis. Bilateral patchy tree in bud opacities and peribronchial nodular opacities; right lung upper lobe peribronchial nodule decreased	Cylindrical bronchiectasis with diminished mucoid impaction and tree-in-bud opacities.	Minimal residual bronchiectasis. Minimal tree in bud opacities within the periphery of the upper lobes, decreasing since prior examination.

## Discussion

Like in APECED, patients with thymoma have an abnormal thymic microenvironment leading to decreased AIRE expression, impaired negative selection of T-cells, and an enrichment of anti-cytokine antibodies against type-1 interferons, particularly anti-IFN-ω, that collectively result in a broad spectrum of autoimmune manifestations [13]. Although we had previously reported patients with thymoma-associated and APECED-associated autoimmune pneumonitis display common clinical, radiographic, histological, and bronchial-directed autoantibodies (BPIFB1 and KCNRG), there has been no published literature on how to treat thymoma-associated pneumonitis. In both cases, our patients initially presented with mycobacterial disease in the setting of chronic, uncontrolled airway inflammation that persisted despite anti-mycobacterial therapy, which we have also observed in some patients with uncontrolled APECED-associated autoimmune pneumonitis [5, 14, 15]. Importantly, the histological presentation of autoimmune pneumonitis differs from that of patients without thymoma infected with NTM. While both patient groups may have chronic inflammation of the small airways, non-thymoma patients with NTM infection display necrotizing or non-necrotizing granulomas surrounded by giant cells, a finding absent in our thymoma patients [16]. Furthermore, Patient 1 had biopsy-proven recrudescence of autoimmune pneumonitis in the absence of mycobacterial disease. Initiation of lymphocyte-directed immunosuppression not only remitted thymoma-associated autoimmune pneumonitis in both patients but also ameliorated Patient 2’s other autoimmune features of hepatitis and alopecia.

In summary, these cases illustrate the common clinical, radiographic, histological, and autoantibody features of autoimmune pneumonitis caused by APECED and thymoma. Beyond performing a chest CT, measurement of autoantibodies against BPIFB1 and KCNRG is a valuable diagnostic tool and should be considered as a screening modality in patients with thymoma and radiographic evidence of lung disease and/or chronic respiratory symptoms. Prospective evaluation in consecutive patients with thymoma-associated autoimmune pneumonitis will be important to investigate the sensitivity and specificity of these autoantibodies for this entity. In addition, these cases provide proof-of-concept that lymphocyte-directed immunomodulation may remit thymoma-associated autoimmune pneumonitis and hepatitis. Importantly, they expand upon the previously reported therapeutic role of AZA and rituximab in APECED-associated autoimmune pneumonitis and CVID-associated granulomatous and lymphocytic interstitial lung disease. Additional longitudinal follow-up of our patients will be needed to establish the durability of the observed clinical and radiographic responses. Evaluation of AZA and rituximab-based therapy in a larger number of patients will be required to establish the safety and efficacy of this immunomodulatory therapy in the management of autoimmune lung disease in thymoma patients.

## Data Availability

No datasets were generated or analysed during the current study.
